# Differential diagnosis of uterine adenosarcoma: identification of *JAZF1-BCORL1* rearrangement by comprehensive cancer genomic profiling

**DOI:** 10.1186/s13000-022-01279-4

**Published:** 2023-01-13

**Authors:** Chie Hasegawa, Kota Washimi, Yukihiko Hiroshima, Rika Kasajima, Keiji Kikuchi, Tsuguto Notomi, Hisamori Kato, Toru Hiruma, Shinya Sato, Yoichiro Okubo, Emi Yoshioka, Kyoko Ono, Yohei Miyagi, Tomoyuki Yokose

**Affiliations:** 1 Department of Pathology, Kanagawa Cancer Center, 2-3-2 Nakao Asahi-ku, Yokohama, Kanagawa 241-8515 Japan; 2 Division of Advanced Cancer Therapeutics, Kanagawa Cancer Center Research Institute, Yokohama, Kanagawa Japan; 3 Center for Cancer Genome Medicine, Kanagawa Cancer Center, Yokohama, Kanagawa Japan; 4 Division of Molecular Pathology and Genetics, Kanagawa Cancer Center Research Institute, Yokohama, Kanagawa Japan; 5 Department of Gynecology, Kanagawa Cancer Center, Yokohama, Kanagawa Japan; 6 Department of Musculoskeletal Tumor Surgery, Kanagawa Cancer Center, Yokohama, Kanagawa Japan

**Keywords:** Uterine adenosarcoma, Comprehensive genomic profiling, *JAZF1-BCORL1*

## Abstract

**Background:**

Uterine adenosarcoma is a rare malignant tumor that accounts for 8% of all uterine sarcomas, and less than 0.2% of all uterine malignancies. However, it is frequently misdiagnosed in clinical examinations, including pathological diagnosis, and imaging studies owing to its rare and non-specific nature, which is further compounded by the lack of specific diagnostic markers.

**Case presentation:**

We report a case of uterine adenosarcoma for which a comprehensive genomic profiling (CGP) test provided a chance to reach the proper diagnosis. The patient, a woman in her 60s with a history of uterine leiomyoma was diagnosed with an intra-abdominal mass post presentation with abdominal distention and loss of appetite. She was suspected to have gastrointestinal stromal tumor (GIST); the laparotomically excised mass was found to comprise uniform spindle-shaped cells that grew in bundles with a herringbone architecture, and occasional myxomatous stroma. Immunostaining revealed no specific findings, and the tumor was diagnosed as a spindle cell tumor/suspicious adult fibrosarcoma. The tumor relapsed during postoperative follow-up, and showed size reduction with chemotherapy, prior to regrowth. CGP was performed to identify a possible treatment, which resulted in detection of a *JAZF1-BCORL1* rearrangement. Since the rearrangement has been reported in uterine sarcomas, we reevaluated specimens of the preceding uterine leiomyoma, which revealed the presence of adenosarcoma components in the corpus uteri. Furthermore, both the uterine adenosarcoma and intra-abdominal mass were partially positive for CD10 and BCOR staining.

**Conclusion:**

These results led to the conclusive identification of the abdominal tumor as a metastasis of the uterine adenosarcoma. The *JAZF1-BCORL1* rearrangement is predominantly associated with uterine stromal sarcomas; thus far, ours is the second report of the same in an adenosarcoma. Adenosarcomas are rare and difficult to diagnose, especially in atypical cases with scarce glandular epithelial components. Identification of rearrangements involving *BCOR* or *BCORL1*, will encourage BCOR staining analysis, thereby potentially resulting in better diagnostic outcomes. Given that platinum-based chemotherapy was proposed as the treatment choice for this patient post diagnosis with adenosarcoma, CGP also indirectly contributed to the designing of the best-suited treatment protocol.

## Background

Uterine adenosarcoma is a biphasic tumor comprising benign glandular epithelial and sarcomatous components that form lobulated polyp-like lesions [1]. It is a rare malignant tumor that accounts for 8% of all uterine sarcomas and less than 0.2% of all uterine malignancies [2]. The disease has a lower age of onset than that of carcinosarcomas; in fact, a study in which 100 patients were analyzed revealed an age distribution of 14‒89 years, with a median age of onset of 58 years [3]. Vascular invasion, differentiation into rhabdomyosarcoma, and overgrowth of the sarcoma components have been reported to contribute to poor prognosis [4]. The disease is frequently misdiagnosed in clinical examinations and imaging studies owing to its rare and non-specific nature, which is further compounded by the lack of specific diagnostic tests [5]. Comprehensive genomic profiling (CGP) of tumors received pharmaceutical approval in Japan in 2018. The tests were covered by insurance in 2019 for patients with advanced disease progression, who had already received all available standard treatments without success. In sharp contrast to conventional companion diagnostics, CGP examines a substantial number of genetic mutations that may or may not have a direct connection to the treatment protocol being followed. The results of these tests are comprehensively evaluated by a board of experts in chemotherapy, genomic medicine, and diagnostic pathology to determine the optimal treatment strategy best suited to the patient. Herein, we describe a case of advanced abdominal mass, which was diagnosed as metastasis of adenosarcoma of the uterus using CGP.

## Case presentation

A woman in her 60s with a body mass index (BMI) of 22.7 visited her local doctor with complaints of abdominal distension and loss of appetite. She had undergone total hysterectomy and bilateral adnexectomy for uterine leiomyoma at another hospital 6 years prior. In terms of family history, her father had suffered from stomach cancer and her sister had been diagnosed with uterine leiomyoma.

**Fig. 1:**
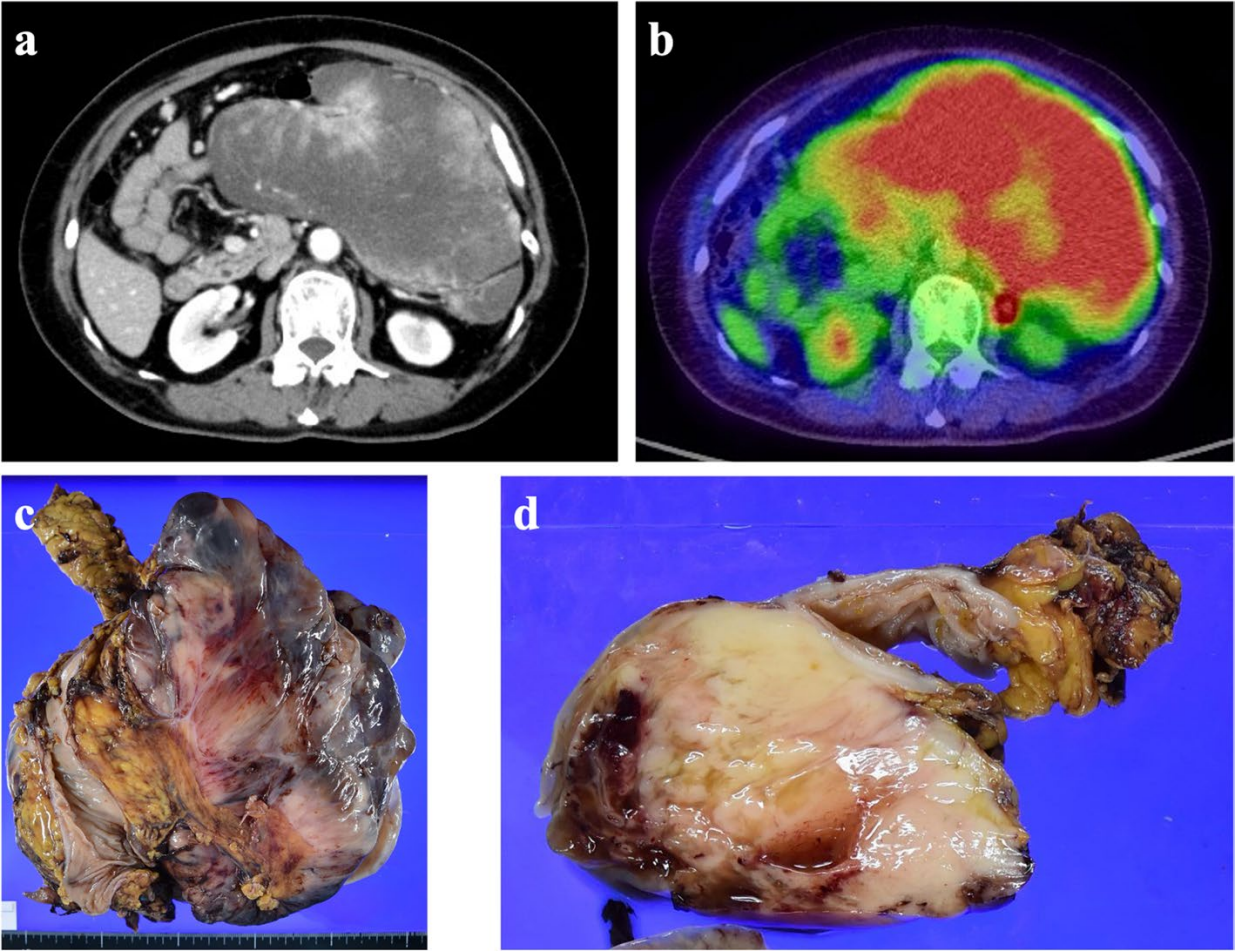
**a** Contrast-enhanced CT findings revealed a large submucosal mass approximately 180 mm in length, contiguous with the transverse to descending colon. Heterogeneous contrast images were visible within the mass. **b** PET-CT demonstrated FDG accumulation (SUVmax: 9.42) within the mass, with no evidence of obvious abnormal accumulation elsewhere. **c** The resected intra-abdominal tumor was 260 × 250 × 120 mm in size and weighed approximately 3.5 kg. **d** The cut surface of the mass was grayish-white, with a circumscribed surface, and an extramural growth from the transverse colon. Mucinous degeneration was observed in certain areas along with a certain degree of bleeding

Upper endoscopy revealed an extramural gastric mass, and computed tomography (CT) demonstrated a left upper abdominal mass. The patient was referred to the Kanagawa Cancer Center for further evaluation and treatment. The differential diagnoses included gastrointestinal stromal tumor (GIST), lymphoma, and peritoneal cancer, and repeat CT scans were performed for confirmation. A large mass with a longitudinal diameter of approximately 180 mm in axial section was observed on the midline, with a slight deviation toward the left abdomen (Fig. 1a). The tumor was contiguous with the transverse colon, without luminal lesions. Positron emission tomography demonstrated 18 F-fluorodeoxyglucose (FDG) accumulation in the mass, with a maximum standardized uptake value (SUVmax) of 9.42. No evidence of space-occupying lesions was found in any other part of the abdomen or in other organs (Fig. 1b).

A transverse colon biopsy was performed via colonoscopy. Histologically, the tumor was abundant in spindle-shaped cells, which were negative for c-kit, DOG-1, CD31, CD34, desmin, myogenin, S100 protein, MDM2, and CDK4, with a Ki-67 index of 20% as observed via immunohistochemical analysis. The pathological diagnosis was limited to a descriptive spindle cell tumor, which led to an open lumpectomy being performed 33 days after the CT scan.

**Fig. 2:**
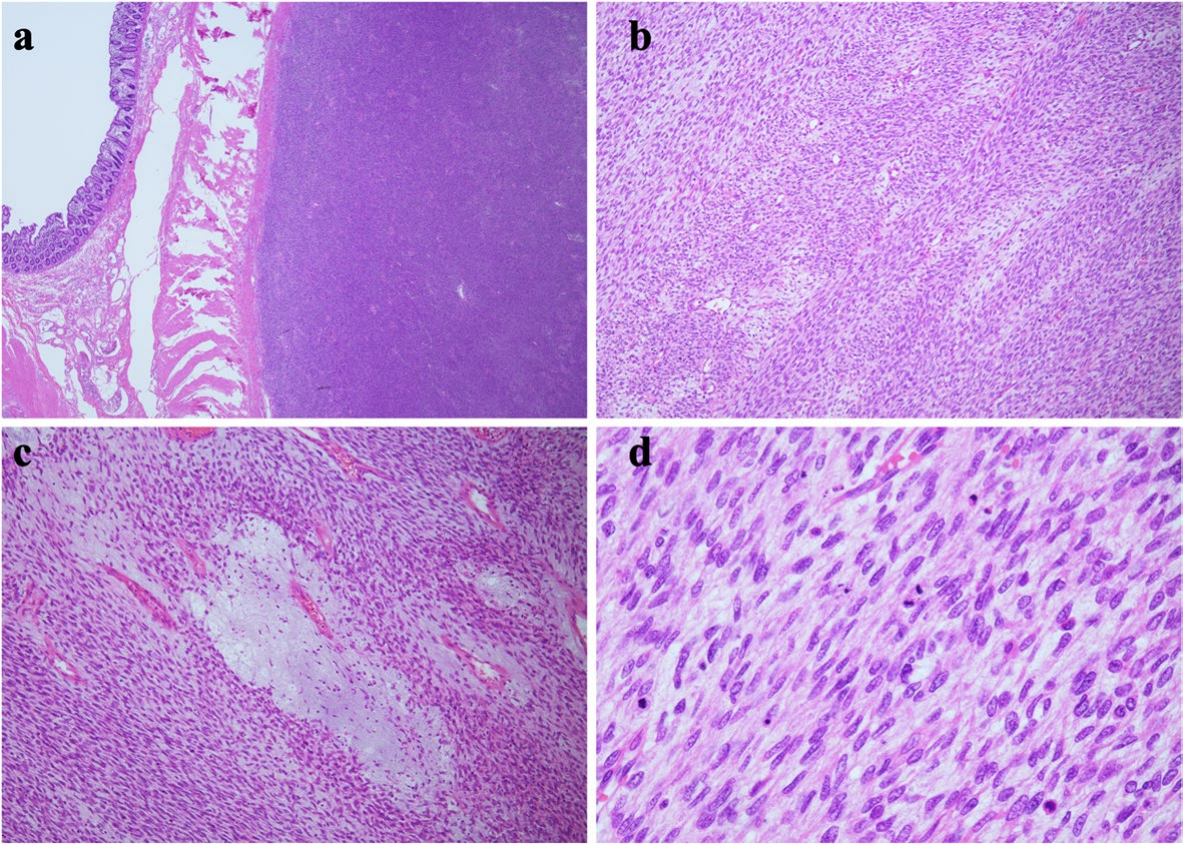
**a** A well-defined extramural mass attached to the transverse colon. **b** Dense proliferating spindle-shaped cells with bundle and herringbone architecture. **c** Hypervascularization with scattered edematous stroma. **d** Spindle-shaped cells with relatively uniform nuclei and prominent mitotic figures

The resected tumor was 260 × 250 × 120 mm in size, weighed approximately 3.5 kg, and was grossly located in the submucosa of the transverse colon (Fig. 1c). The tumor was solid, well-demarcated, grayish-white, and partially hemorrhagic with some degree of mucinous degeneration (Fig. 1d). The mass was located outside the bowel wall (Fig. 2a), and histologically comprised uniform spindle-shaped tumor cells that proliferated in bundles or herringbone patterns (Fig. 2b). Sparse and dense cell areas were intermingled with edematous stroma to some extent (Fig. 2c). Mitotic figures were abundant with a frequency of 39/10 High Power Fields (HPFs) (Fig. 2d), and necrosis was commonly observed. The spindle-shaped tumor cells stained positive for CD99 and were weakly positive for CyclinD1 and CD10 as observed via immunohistochemical analysis. Furthermore, the cells stained negative for cytokeratin AE1/AE3, CAM5.2, EMA, CDK4, MDM2, DOG-1, CD31, CD34, SMA, desmin, myogenin, S100 protein, synaptophysin, STAT6, MUC4, ER, and PgR. Additionally, INI1 expression was confirmed and the Ki-67 index was found to be approximately 30%. These findings resulted in the tumor being diagnosed as a spindle cell sarcoma that was highly suggestive of adult fibrosarcoma of intra-abdominal origin.

CT revealed recurrence of the tumor as evidenced by the presence of multiple masses in the abdominal cavity 4 months post tumor removal. Given the challenge associated with radical surgical removal of the tumors, chemotherapy with doxorubicin and ifosfamide was selected as the treatment of choice based on the histological diagnosis of fibrosarcoma. The recurrent masses were remarkably smaller, and chemotherapeutic protocol was adjudged to have resulted in a complete response post administration of the fifth course (14 months postoperatively). Consequently, chemotherapy was temporarily halted; however, CT indicated re-expansion of the intra-abdominal mass 4 months later (18 months postoperatively). Although monotherapy with doxorubicin was initiated, the response was unsatisfactory and the cancer was categorized as a progressive disease (PD). Chemotherapy was subsequently switched to ifosfamide and etoposide, which initially achieved a partial response (PR). However, on account of enlargement of the pelvic mass after the 7th course, the cancer remained a PD. A CGP test was ultimately performed to explore possible treatment options.

Formalin-fixed and paraffin-embedded tissue from the initially excised abdominal tumor was subjected to the CGP test, Foundation One® CDx (Chugai Pharmaceutical Co. Ltd., Japan), which assesses the entire coding sequence of 315 cancer-related genes plus introns from 28 genes often rearranged or altered in cancer. The test was performed successfully using the 96.7% pure sample comprising 80% tumor nuclei. The tumor mutational burden (TMB) was estimated to be 1.26 mutations/Mb, and the microsatellite status (MSS) was stable. The CGP test identified four small variants, including three single-nucleotide polymorphisms (SNPs) and a variant of uncertain significance (VUS). A single copy number loss, corresponding to exons 3 to 5 of *CDKN2A*, was identified as a pathogenic alteration that resulted in loss of function of the tumor suppressor. Additionally, a high confidence rearrangement between *JAZF1* and *BCORL1* with 276 supporting-read pairs was identified. This rearrangement was deduced to produce truncated BCORL1, albeit, with a degree of uncertainty on account of the lack of RNA-based analysis. Considering that *JAZF1-BCORL1* rearrangements have been previously reported in uterine stromal sarcoma, we decided to re-evaluate surgical specimens obtained post the simple total hysterectomy performed at the other hospital for the preceding uterine leiomyoma in this patient.

**Fig. 3:**
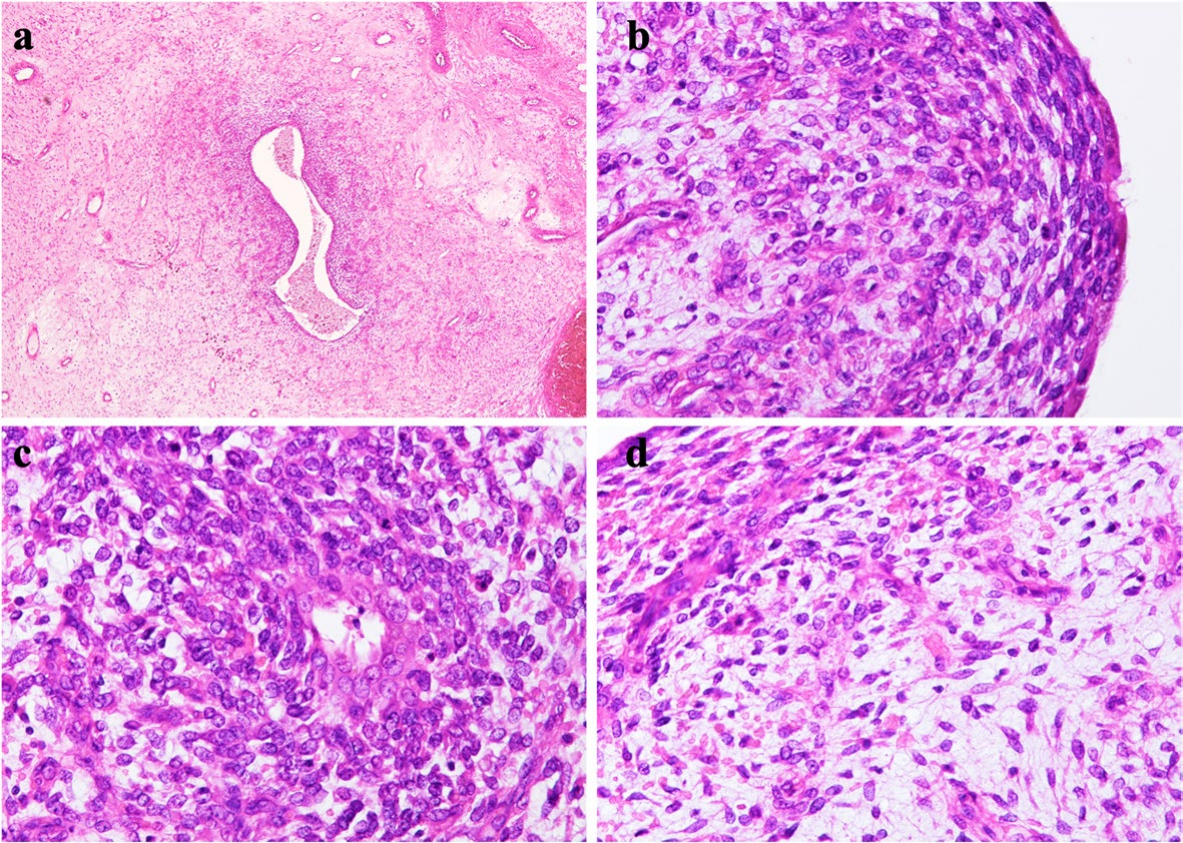
**a** Tubular glandular lumen with high cell density around the gland. **b** Increased cell density under the epithelium. **c** Stromal cells showing nuclear enlargement with a high N/C ratio and scattered mitotic figures. **d** Edematous stroma observed in some areas

**Fig. 4:**
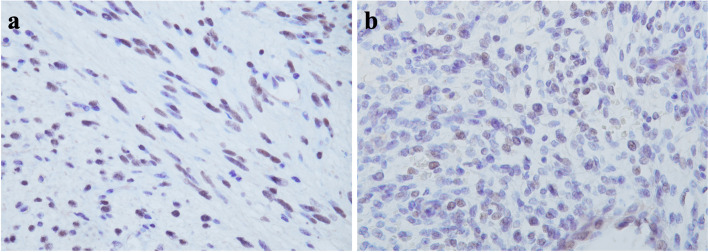
**a** BCOR staining of the intra-abdominal tumor. **b** BCOR staining of uterine adenosarcoma cells

The hysterectomy specimen showed dense proliferation of uniform spindle cells in the stroma immediately below the glandular epithelium, without nuclear atypia in addition to the leiomyoma (Fig. 3a, b). Leaf-like architecture of growth with intraglandular polypoid projections of the stroma was observed in certain areas. The atypical stromal cells had irregular oval to spiniform nuclei with mitotic figures at a frequency of approximately 5/10 HPFs (Fig. 3c). The proliferating stromal spindle cells were densely packed immediately below the benign glandular epithelium with an edematous stroma (Fig. 3d), and were positive for PgR and partially positive for desmin, CyclinD1, ER, WT-1, and CD10. Furthermore, these cells were generally SMA-negative, with a Ki-67 index of 20%. These findings were consistent with a diagnosis of uterine adenosarcoma. Since BCOR was shown to be strongly expressed in adenosarcoma carrying the *JAZF1-BCORL1* fusion gene, we examined the expression of BCOR in our case by immnohistochemistry using an anti-BCOR monoclonal antibody (clone C-10, Santa Cruz Biotechnology, Dallas, TX) [6]. Both spindle cells in the corpus uteri and the intra-abdominal mass showed partial nuclear positivity for BCOR (Fig. 4a, b), suggesting that the intra-abdominal mass was a metastatic tumor derived from the preceding uterine adenosarcoma that may carry the *JAZF1-BCORL1* fusion gene. Given that uterine stromal sarcomas often harbor a *JAZF1-BCORL1* rearrangement, platinum-based chemotherapy, which had not been used before, was considered for this patient at the hospital’s review meeting [7, 8]. However, the same could not be implemented on account of recurrent enlarged abdominal tumors with pulmonary metastasis, which resulted in rapid deterioration of the patient’s general condition, and adoption of a non-aggressive treatment approach.

## Discussion and conclusions

Herein, we present a case of uterine adenosarcoma confirmed to be metastasis using CGP examination. Uterine adenosarcoma is a rare tumor that is commonly diagnosed histopathologically, and its diagnosis is often complicated, especially in cases with atypical histological appearance [2]. Although the patient had previously been treated surgically for a uterine leiomyoma, a differential diagnosis of metastatic uterine sarcoma was not considered until results of the CGP test were obtained, in which a characteristic gene rearrangement was identified. This further led to the consideration of platinum-based chemotherapy for the patient.

Risk factors for uterine adenosarcomas include a history of pelvic irradiation, excess estrogen, tamoxifen treatment, obesity, diabetes, endometriosis, and adenomyosis [9, 10]. The present patient, with a BMI of 22.7, was not obese or diabetic, and had no history of tamoxifen treatment. Sarcomatous overgrowth, myometrial invasion, size, mitosis, age, FIGO stage, resection status, necrosis, cellular atypia, heterologous elements, and rhabdomyosarcoma constitute the prognostic factors for uterine adenosarcoma [2]. In this case, there was no obvious differentiation into rhabdomyosarcoma in either the uterine lesion or in any markedly prominent sarcomatoid area. Endometriosis and adenomyosis have been previously reported in the context of adenosarcoma but could not be confirmed in this case despite rigorous investigations. The diagnosis was further complicated because of the presence of an adjacent uterine leiomyoma.

The immunophenotype of adenosarcoma spindle cells has been reported to resemble that of endometrial stromal tumors that are often positive for CD10, ER, and PgR expression, which tends to disappear in cases with sarcomatous overgrowth [11]. In the present case, while the spindle cells in the uterine adenosarcoma and intra-abdominal tumor were weakly positive for CD10, only the former was partially positive for ER and PgR. The Ki-67 index was approximately 20% and 30% in the uterine adenosarcoma and intra-abdominal tumor, respectively, and a Ki-67 index > 30% in adenosarcomas has been reported to be associated with poor prognosis [12]. The Ki-67 index and loss of ER/PgR expression in the present case was suggestive of an aggressive abdominal metastatic tumor that may correspond to a sarcomatous overgrowth lesion. Both tumors in the patient were partially positive for BCOR. A similar observation was reported in a case of uterine adenosarcoma that harbored a *JAZF1-BCORL1* rearrangement, lending credence to the possible utilization of BCOR immunostaining as a useful tool for the diagnosis of this disease [6].

*JAZF1-BCORL1* rearrangement is a genetic abnormality that is frequently found in uterine stromal sarcomas [13] and to the best of our knowledge, ours is only the second reported adenosarcoma case with this rearrangement [6]. *BCORL1* is a homologous transcriptional co-repressor of *BCOR* and plays key roles in transcriptional regulation, since both interchangeably participate in the polycomb repressive complex 1 (PRC1) [14]. *JAZF1-BCORL1*, *EP300-BCORL1*, internal *BCORL1* rearrangement, inactivating *BCORL1* mutations, and homozygous *BCORL1* deletions have been reported in uterine stromal sarcomas and are associated with aggressive clinical behavior [13]. Furthermore, in addition to endometrial stromal sarcomas, high-grade uterine sarcomas and myxoid uterine leiomyosarcomas have also been reported to harbor *BCORL1* mutations. Approximately half of all sarcomas with *BCORL1* mutations demonstrate either CDK4 amplification or *CDKN2A* deficiency [13]. Considering that a similar loss of *CDKN2A* was observed in the CGP test in the present case, the clinical utilization of CDK4 inhibitors should be given due consideration in the future.

Approximately 25% of high-grade endometrial stromal sarcomas with *BCORL1* mutations are reported to be misdiagnosed as leiomyosarcoma [13]. While our findings imply that BCOR immunostaining may be useful in ensuring an accurate diagnosis in adenosarcomas with *BCORL1* mutations, conclusive evidence will require evaluation of several more cases. Although performance of genetic tests in all uterine stromal tumors during routine diagnostic procedures is impractical, close attention must be paid to CGP tests performed for patients in advanced stages, which may provide crucial clues that may aid accurate diagnosis, especially in cases of rare tumors, such as metastatic adenosarcoma.

The known repertoire of pathogenic genetic alterations associated with tumors is expanding at an exponential rate, and experts in pathology, genetics, and cancer chemotherapeutics need to make collaborative efforts in order to benefit maximally from results obtained using advanced testing, such as CGP tests. Although the CGP test in the present case did not suggest targeted molecular therapies, the results helped pathologists reach an accurate diagnosis that could be utilized to strategize an optimal treatment regimen tailored specifically to the tumor characteristics.

## Data Availability

The dataset supporting the conclusions of this study is included within the article, and all materials are available upon reasonable request.
